# Protocol for single-cell ATAC sequencing using combinatorial indexing in mouse lung adenocarcinoma

**DOI:** 10.1016/j.xpro.2021.100583

**Published:** 2021-06-03

**Authors:** Isabella Del Priore, Sai Ma, Jonathan Strecker, Tyler Jacks, Lindsay M. LaFave, Jason D. Buenrostro

**Affiliations:** 1 David H. Koch Institute for Integrative Cancer Research, Massachusetts Institute of Technology, Cambridge, MA 02142, USA; 2 Department of Biology, Massachusetts Institute of Technology, Cambridge, MA 02142, USA; 3 Broad Institute of MIT and Harvard, Cambridge, MA 02142, USA; 4 Department of Stem Cell and Regenerative Biology, Harvard University, Cambridge, MA 02138, USA

**Keywords:** Cancer, Genomics, Molecular Biology, Sequencing, Single Cell

## Abstract

Single-cell ATAC sequencing using combinatorial indexing (sciATAC-seq) enables the identification of chromatin accessibility profiles at single-cell resolution with a dual-barcoding approach during transposition and library construction. Unlike commercial droplet-based approaches, sciATAC-seq is a cost-effective, extensible strategy that permits flexibility in the experimental scale via multiplexed barcoding across samples or perturbations. In contrast, droplet-based approaches have higher cell recovery and may be advantageous when cell input is limited. The step-by-step sciATAC-seq protocol described here is amenable to diverse cell types and fixed samples.

For complete details on the use and execution of this protocol, please refer to LaFave et al. (2020).

## Highlights

• Optimized single-cell ATAC-seq protocol using combinatorial indexing
• Flexible platform for epigenomic profiling of cell lines or primary tissues
• Computational framework for initial data processing of sciATAC-seq data

## Graphical abstract

**Figure:**
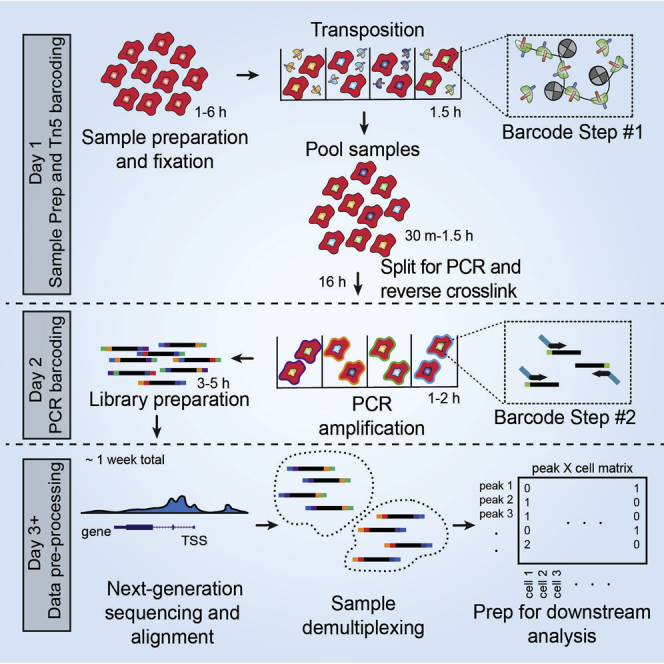

## Before you begin

This optimized sciATAC-seq protocol uses cellular combinatorial indexing (Cusanovich et al., 2015) to assign unique barcodes to samples at transposition and PCR. Individual samples are labeled at transposition followed by pooling and redistribution to 96 well plates for a second barcode step during PCR amplification. Transposition adapters and PCR primers can be prepared ahead of time and stored long-term at −20°C or −80°C. Prior to beginning the protocol, researchers will need to purify Tn5 transposase (Ma et al., 2020, adapted from Picelli et al., 2014) and confirm enzymatic efficiency. Primer sequences for the dual-barcoding approach are found in the key resources table. Some solutions can be prepared in advance and stored as indicated. Throughout the protocol, we provide suggestions for when buffers should be prepared fresh. Refer to the key resources table for a complete list of materials and equipment necessary for this protocol. Researchers will need to optimize dissociation protocols to obtain viable cells for varied tissue types, which are not described in this protocol.

### Anneal transposition adapters

**Timing: [1 h 30 min]**

Mosaic end (ME)-complement and Illumina sequencing adapter oligos are annealed for subsequent loading onto the Tn5 enzyme.

1. Dissolve sciAD1.X and sciAD2.X transposition oligos (see key resources table) to a final concentration of 100 μM in IDTE.

***Note:*** sciAD1.X and sciAD2.X transposition oligos should be ordered as HPLC purified.

***Note:*** In the sciAD1.X nomenclature, X refers to the repertoire of barcodes that can be used at transposition.

2. Combine 13 μL of 100 μM sciAD1.X or 13 μL of sciAD2.X oligos with 13 μL 100 μM ME-Comp oligo, 0.26 μL 1M Tris pH 8.0, and 0.26 μL 5M NaCl to make up 20 individual sci-oligo mixes.

***Note:*** A standard transposition reaction is completed in a 96 well format with 8 sciAD1 and 12 sciAD2 sci-oligo mixes to be prepared.

3. Heat sci-oligo mixes in a thermal cycler at 85°C for 2 min, then slowly cool down to 20°C at a rate of −1°C/min to generate an annealed adapter mix.
4. Mix 25 μL annealed adapter mix with 25 μL cold 100% glycerol and store at −20°C or −80°C until use. See Table 1 for example plate organization of annealed adapters.

**Table 1:** Primer plate for sciAD1 and sciAD2 oligos

		sciAD1.3	sciAD1.4	sciAD1.5	sciAD1.6	sciAD1.7	sciAD1.9	sciAD1.10	sciAD1.11		
sciAD2.1	sciAD2.3	sciAD2.4	sciAD2.6	sciAD2.7	sciAD2.8	sciAD2.11	sciAD2.12	sciAD2.13	sciAD2.14	sciAD2.15	sciAD2.17

***Note:*** Annealed adapters are stable long-term at −20°C or −80°C and can be prepared prior to experiment start. This protocol provides directions for transposition in a 96 well plate format which will require 4 μL of each sciAD1 and 4 μL of each sciAD2 annealed oligo mix per experiment.

### Prepare PCR primer plates

**Timing: [20 min]**

5. Dilute PCR primers to a final concentration of 100 μM in IDTE.
6. Pipette 50 μL of PCR primers into 96 well plates for a working stock. We recommend storage in two 96 well plates (3 rows for sciP1.X and 3 columns sciP2.X). An example of the storage plates is provided (Tables 2 and 3).

**Table 2:** Primer plate for sciP1

sciP1.01	sciP1.02	sciP1.03	sciP1.04	sciP1.05	sciP1.06	sciP1.07	sciP1.08	sciP1.09	sciP1.10	sciP1.11	sciP1.12
											
											
sciP1.13	sciP1.14	sciP1.15	sciP1.16	sciP1.17	sciP1.18	sciP1.19	sciP1.20	sciP1.21	sciP1.22	sciP1.23	sciP1.24
											
											
sciP1.25	sciP1.26	sciP1.27	sciP1.28	sciP1.29	sciP1.30	sciP1.31	sciP1.32	sciP1.33	sciP1.34	sciP1.35	sciP1.36

**Table 3:** Primer plate for sciP2

sciP2.01				sciP2.09				sciP2.17			
sciP2.02				sciP2.10				sciP2.18			
sciP2.03				sciP2.11				sciP2.19			
sciP2.04				sciP2.12				sciP2.20			
sciP2.05				sciP2.13				sciP2.21			
sciP2.06				sciP2.14				sciP2.22			
sciP2.07				sciP2.15				sciP2.23			
sciP2.08				sciP2.16				sciP2.24

***Note:*** In one experiment, up to 9 plates of PCR can be processed. Each PCR plate results in 9,216 combinations (12 sciAd1 ∗ 8 sciAd2 ∗ 12 sciP1 ∗ 8 sciP2 = 9,216 combinations) at the second round of barcoding. Each PCR plate yields on average 2,000 epigenomic profiles. Across 9 plates, up to 18,000 (2,000∗9) single cell profiles could be generated. Upfront the researcher should decide how many cells they need at the end of the experiment.

### Transposome preparation

**Timing: [1 week]**

Tn5 transposase should be prepared (Picelli et al., 2014, Ma et al., 2020) as described below, which will be used at the Tn5 assembly and transposition steps.

7. Perform bacterial transformation of pTXB1-Tn5 expression vector (Picelli et al., 2014) into C3013 cells and incubate for 16 h at 37°C on Luria Broth (LB) plates with 100 μg/mL ampicillin.
8. Inoculate a 10 mL starter culture of LB media with 100 μg/mL ampicillin and incubate at 37°C in a bacterial shaker at 200 rpm for 16 h.
9. Add 10 mL of culture to a new flask of 1 L LB media with 100 μg/mL ampicillin pre-added.
10. Incubate in a shaker at 37°C at 200 rpm until the Optical Density (OD) reaches 0.6 using a spectrophotometer.

***Note:*** This should take approximately 3–4 h but can vary based on the bacterial shaker used.

11. Chill culture on ice for 30 min once OD 0.6 is reached.
12. Add IPTG at a final concentration of 0.25 mM to induce Tn5 expression, then incubate the culture at 18°C while shaking at 200 rpm for 16 h.

***Note:*** IPTG should be prepared fresh prior to use.

13. Collect pellet by centrifugation at 4000 × *g* for 15 min at 4°C.
14. Remove the supernatant and flash freeze the pellet at −80°C for 30 min or longer. Resuspend the frozen pellet in 40 mL chilled HEGX buffer with 1× Roche Complete EDTA-free protease inhibitor tablet.

***Optional:*** Add 10 μL of Benzonase nuclease to the resuspended pellet.

15. Lyse cells using a Bioruptor Plus sonicator with 50% duty cycles, keeping cells on ice until sufficiently lysed. Alternatively, use a French press or microfluidizer at appropriate settings to lyse the cells.
16. Centrifuge sonicated lysate at 30,000 × *g* for 20 min at 4°C.
17. Add 1.05 mL of 10% PEI (pH 7) dropwise to the stirring lysate solution and centrifuge for 10 min at 9000 × *g* at 4°C to remove precipitated *E. coli* DNA.
18. Prepare an Econo-Pac Chromatography Column by packing a 2 mL aliquot of a chitin slurry resin into a disposable column.
19. Prepare HEGX buffer.
20. Wash column with 30 mL HEGX buffer.
21. Slowly add the soluble fraction to the chitin resin, then incubate on a rotator at 4°C for at least 8 h.
22. Wash the column thoroughly with 40 mL HEGX buffer.
23. Elute chitin slurry with 10 mL of elution buffer (HEGX supplemented with 100 mM DTT) on a rotator at 4°C for 48 h.
24. Collect eluate and dialyze twice in 500 mL of Tn5 Dialysis Buffer.
25. Determine the concentration of Tn5 using an A280 measurement (an A280 of 1.0 equals 0.616 mg/mL or 11.56 mM Tn5). Concentrate dialyzed protein using an Amicon Ultra-4 Centrifugal Filter Units 30 K if required to an A280 of 2.5.

**Figure 1:**
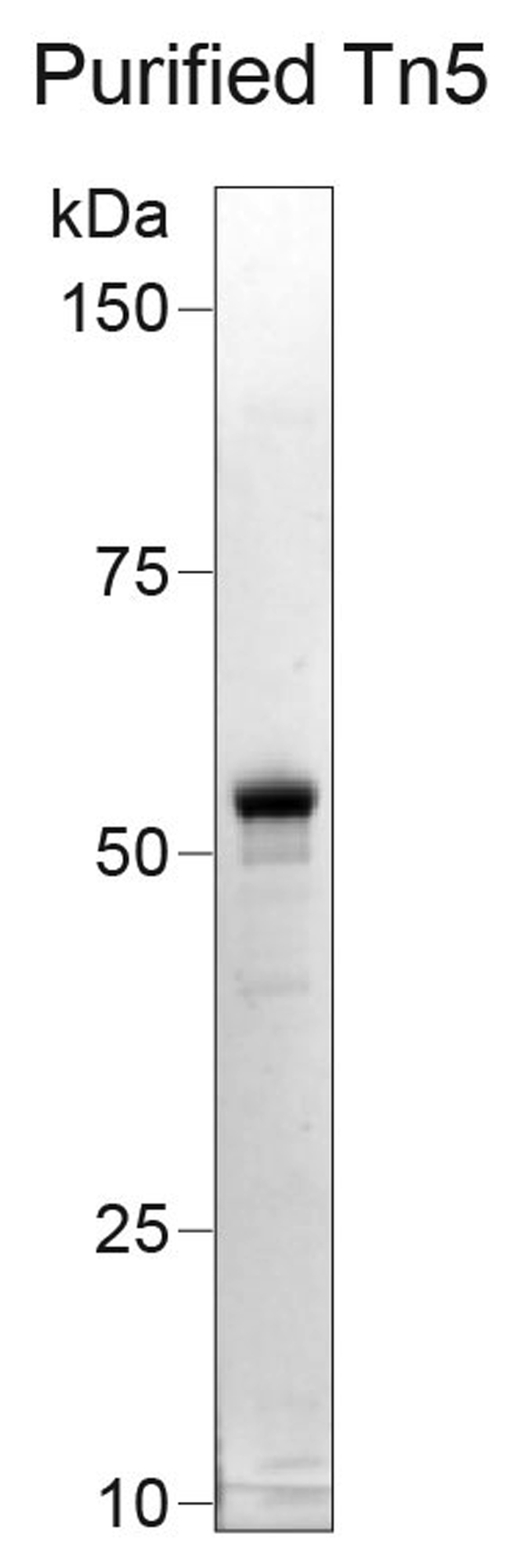
Expected molecular weight of purified Tn5 enzyme Tn5 prep run on SDS-Page to demonstrate purity and appropriate molecular weight.

***Note:*** As an additional quality control, purified Tn5 enzyme can be run on an SDS-page gel to confirm appropriate molecular weight and purity (Figure 1).

26. Add sterile 100% glycerol 1:1 to create a final 50% glycerol stock of the purified protein.
27. Confirm activity of the transposase by performing a comparison to bulk ATAC sequencing experiment with the commercially available TDE1 transposome (Buenrostro et al., 2013). Load purified transposase with a pair of adapters as described later in this protocol. Perform tagmentation experiment on 50 ng purified genomic DNA, such as commercially available HeLa genomic DNA.
28. Quantify the number of cycles to reach 1/3 of the maximum fluorescent intensity (Buenrostro et al., 2015) and use these values to compare Tn5 efficiency (Troubleshooting 1).
29. Dilute prepared Tn5 to the appropriate concentration where transposition efficiency is comparable to that of the commercially available Nextera TDE1 transposome using Dilution Buffer.

## Key resources table

**Table:** 

REAGENT or RESOURCE	SOURCE	IDENTIFIER
**Antibodies**		

CD11B-APC	eBioscience	Cat# 7-0112-82;RRID:AB_469344
TER119-APC	eBioscience	Cat# 557909;RRID:AB_398635
CD45-APC	BD Biosciences	Cat# 559864;RRID:AB_398672
CD31-APC	BioLegend	Cat# 102510;RRID:AB_312917

**Bacterial and virus strains**		

T7 Express *lysY/Iq* Competent *E. coli* (High Efficiency) (C3013 cells)	New England Biolabs	C3013I

**Chemicals, peptides, and recombinant proteins**		

DPBS, 1× without calcium and magnesium	VWR Scientific Inc.	Cat# 21-031-CV
DMEM with L-glutamine	VWR Scientific Inc.	Cat# 10-013-CV (45000-304)
S-MEM	Life Technologies	Cat# 11380-037
ACK Lysing Buffer	Thermo Fisher Scientific	Cat# a10492-01
16% Methanol-free formaldehyde	Thermo Fisher Scientific	Cat# 28908
Glycine	VWR Scientific Inc.	Cat# 97061-128
Tris (1M). pH 8.0	Thermo Fisher Scientific	Cat# AM9855G
Bovine Albumin Fraction V (7.5% solution)	Thermo Fisher Scientific	Cat# 15260037
NaCl, 5M	Thermo Fisher Scientific	Cat# AM9759
0.5M EDTA pH 8.0	Thermo Fisher Scientific	Cat# 15575-020
DTT (dithiothreitol)	Thermo Fisher Scientific	Cat# R0861
NP-40 Surfact-Amps Detergent Solution	Thermo Scientific Nalgene	Cat# 28324
Tris Acetate Buffer 0.2M, pH 7.8	bioWORLD	Cat# 40120265-3
Potassium acetate solution	Sigma-Aldrich	Cat# 95843
Magnesium acetate solution	Sigma-Aldrich	Cat# 63052
Sequencing-grade dimethylformamide (DMF)	Thermo Fisher Scientific	Cat# 20673
Halt Protease Inhibitor Cocktail (100×)	Thermo Fisher Scientific	Cat# 78430
MgCl_2_ (1M)	Thermo Fisher Scientific	Cat# AM9530G
UltraPure 1M Tris-HCl Buffer, pH 7.5	Thermo Fisher Scientific	Cat# 15567027
Sodium Dodecyl Sulfate	Bio-Rad	Cat# 161-0302
Proteinase K, recombinant, PCR grade solution	Sigma-Aldrich	Cat# 3115828001
Tween(R)20, SigmaUltra	Sigma-Aldrich	Cat# P7949-100ML
SYBR Fast 2× MM LC480	Kapa Biosystems	Cat# KK4611
SYBR Green I Nucleic Acid Gel Stain	Thermo Fisher Scientific	Cat# S7563
Unloaded Tn5	This paper	(Ma et al., 2020)
pTXB1-Tn5 expression vector	N/A	(Picelli et al., 2014)
Triton X-100	Sigma-Aldrich	Cat# T9284
IPTG	Sigma-Aldrich	Cat# 15502
cOmplete, EDTA-free Protease Inhibitor Cocktail	Millipore Sigma	Cat# 5056489001
Benzonase nuclease	Sigma-Aldrich	Cat# E1014
IDTE pH 8.0 (1× TE solution)	Integrated DNA Technologies	Cat# 11-05-01-09
Luria Broth Base (Miller’s LB Broth Base) (LB media)	Invitrogen	Cat# 12795-084
Ampicillin	Thermo Fisher Scientific	Cat# BP1760-25
HEPES buffer	Thermo Fisher Scientific	Cat# 15630-080
Glycerol	Sigma-Aldrich	Cat# G9012-2L

**Critical commercial assays**		

Trypan Blue Solution, 0.4%	Thermo Fisher Scientific	Cat# 15250061
NEBNext High-Fidelity 2× PCR Master Mix	New England Biolabs (NEB)	Cat# M0541L
MinElute PCR Purification Kit	QIAGEN	Cat# 28006
KAPA Library Quant for Illumina Sequencing Platforms	Kapa Biosystems	Cat# KK4824
FlashGel^TM^ DNA Cassettes	Lonza Bio	Cat# 57031
Amicon Ultra-4 Centrifugal Filter Units 30K	Millipore Sigma	Cat# UFC803024
Chitin resin	New England Biolabs (NEB)	Cat# S6651S
Econo-Pac Chromatography Columns	Bio-Rad	Cat# 7321010
Illumina Tagment DNA Enzyme and Buffer	Illumina	Cat# 20034197
NextSeq 500/550 High Output Kit v2	Illumina	Cat# FC-404-2002
Lung Dissociation Kit, mouse	Miltenyi Biotec	Cat# 130-095-927

**Experimental models: Cell lines**		

HeLa genomic DNA	New England Biolabs (NEB)	Cat# N4006S

**Experimental models: Organisms/strains**		

KP mouse	Jackson et al., 2001; Jacks et al., 2005	stock 008179, stock 008462
Tomato mouse (Ai9)	Jackson Laboratory	stock 007905

**Oligonucleotides**		

Primer Table		
sciAD1.3_TATCCTCT: CGGAGCTTTGCTAAC GGTCGTATCCTCTTCGTCGGCAGCGTCAGA TGTGTATAAGAGACAG	IDT	This paper
sciAD1.4_AGAGTAGA: CGGAGCTTTGCTA ACGGTCGAGAGTAGATCGTCGGCAGCGT CAGATGTGTATAAGAGACAG	IDT	This paper
sciAD1.5_GTAAGGAG: CGGAGCTTTGCTA ACGGTCGGTAAGGAGTCGTCGGCAGCGT CAGATGTGTATAAGAGACAG	IDT	This paper
sciAD1.6_ACTGCATA: CGGAGCTTTGCTAA CGGTCGACTGCATATCGTCGGCAGCGTCA GATGTGTATAAGAGACAG	IDT	This paper
sciAD1.7_AAGGAGTA: CGGAGCTTTGCTA ACGGTCGAAGGAGTATCGTCGGCAGCGTC AGATGTGTATAAGAGACAG	IDT	This paper
sciAD1.9_TGGAAATC: CGGAGCTTTGCTAA CGGTCGTGGAAATCTCGTCGGCAGCGTCA GATGTGTATAAGAGACAG	IDT	This paper
sciAD1.10_AACATGAT: CGGAGCTTTGCTAA CGGTCGAACATGATTCGTCGGCAGCGTCA GATGTGTATAAGAGACAG	IDT	This paper
sciAD1.11_TGATGAAA: CGGAGCTTTGCTAA CGGTCGTGATGAAATCGTCGGCAGCGTCA GATGTGTATAAGAGACAG	IDT	This paper
sciAD2.1_TAAGGCGA: CTTACGGATGTTGC ACCAGCTAAGGCGAGTCTCGTGGGCTCGG AGATGTGTATAAGAGACAG	IDT	This paper
sciAD2.3_AGGCAGAA: CTTACGGATGTTGC ACCAGCAGGCAGAAGTCTCGTGGGCTCGG AGATGTGTATAAGAGACAG	IDT	This paper
sciAD2.4_TCCTGAGC: CTTACGGATGTTGC ACCAGCTCCTGAGCGTCTCGTGGGCTCGG AGATGTGTATAAGAGACAG	IDT	This paper
sciAD2.6_TAGGCATG: CTTACGGATGTTGC ACCAGCTAGGCATGGTCTCGTGGGCTCGG AGATGTGTATAAGAGACAG	IDT	This paper
sciAD2.7_CTCTCTAC: CTTACGGATGTTGC ACCAGCCTCTCTACGTCTCGTGGGCTCGG AGATGTGTATAAGAGACAG	IDT	This paper
sciAD2.8_CAGAGAGG: CTTACGGATGTTG CACCAGCCAGAGAGGGTCTCGTGGGCTC GGAGATGTGTATAAGAGACAG	IDT	This paper
sciAD2.11_AAGAGGCA: CTTACGGATGTTG CACCAGCAAGAGGCAGTCTCGTGGGCTC GGAGATGTGTATAAGAGACAG	IDT	This paper
sciAD2.12_GTAGAGGA: CTTACGGATGTTG CACCAGCGTAGAGGAGTCTCGTGGGCTC GGAGATGTGTATAAGAGACAG	IDT	This paper
sciAD2.13_TGGATCTG: CTTACGGATGTTG CACCAGCTGGATCTGGTCTCGTGGGCTC GGAGATGTGTATAAGAGACAG	IDT	This paper
sciAD2.14_CCGTTTGT: CTTACGGATGTTG CACCAGCCCGTTTGTGTCTCGTGGGCTC GGAGATGTGTATAAGAGACAG	IDT	This paper
sciAD2.15_TGCTGGGT: CTTACGGATGTT GCACCAGCTGCTGGGTGTCTCGTGGGCT CGGAGATGTGTATAAGAGACAG	IDT	This paper
sciAD2.17_GTGTGGTG: CTTACGGATGTT GCACCAGCGTGTGGTGGTCTCGTGGGC TCGGAGATGTGTATAAGAGACAG	IDT	This paper
sciP1.01_TAGATCGC: AATGATACGGCGA CCACCGAGATCTACACTAGATCGCCGGA GCTTTGCTAACGGTCG	IDT	This paper
sciP1.02_CTCTCTAT: AATGATACGGCGAC CACCGAGATCTACACCTCTCTATCGGAG CTTTGCTAACGGTCG	IDT	This paper
sciP1.03_TATCCTCT: AATGATACGGCGAC CACCGAGATCTACACTATCCTCTCGGAG CTTTGCTAACGGTCG	IDT	This paper
sciP1.04_AGAGTAGA: AATGATACGGCGA CCACCGAGATCTACACAGAGTAGACGGA GCTTTGCTAACGGTCG	IDT	This paper
sciP1.05_GTAAGGAG: AATGATACGGCGA CCACCGAGATCTACACGTAAGGAGCGG AGCTTTGCTAACGGTCG	IDT	This paper
sciP1.06_ACTGCATA: AATGATACGGCGA CCACCGAGATCTACACACTGCATACGGA GCTTTGCTAACGGTCG	IDT	This paper
sciP1.07_AAGGAGTA: AATGATACGGCGA CCACCGAGATCTACACAAGGAGTACGGA GCTTTGCTAACGGTCG	IDT	This paper
sciP1.08_CTAAGCCT: AATGATACGGCGA CCACCGAGATCTACACCTAAGCCTCGGA GCTTTGCTAACGGTCG	IDT	This paper
sciP1.09_TGGAAATC: AATGATACGGCGAC CACCGAGATCTACACTGGAAATCCGGAG CTTTGCTAACGGTCG	IDT	This paper
sciP1.10_AACATGAT: AATGATACGGCGAC CACCGAGATCTACACAACATGATCGGAG CTTTGCTAACGGTCG	IDT	This paper
sciP1.11_TGATGAAA: AATGATACGGCGAC CACCGAGATCTACACTGATGAAACGGAG CTTTGCTAACGGTCG	IDT	This paper
sciP1.12_GTCGGACT: AATGATACGGCGA CCACCGAGATCTACACGTCGGACTCGGA GCTTTGCTAACGGTCG	IDT	This paper
sciP1.13_TTTCTAGC: AATGATACGGCGAC CACCGAGATCTACACTTTCTAGCCGGAG CTTTGCTAACGGTCG	IDT	This paper
sciP1.14_TAACCAAG: AATGATACGGCGA CCACCGAGATCTACACTAACCAAGCGG AGCTTTGCTAACGGTCG	IDT	This paper
sciP1.15_GTGTATCG: AATGATACGGCGAC CACCGAGATCTACACGTGTATCGCGGAG CTTTGCTAACGGTCG	IDT	This paper
sciP1.16_TCCATCAA: AATGATACGGCGAC CACCGAGATCTACACTCCATCAACGGAG CTTTGCTAACGGTCG	IDT	This paper
sciP1.17_TTCGTGCA: AATGATACGGCGAC CACCGAGATCTACACTTCGTGCACGGAG CTTTGCTAACGGTCG	IDT	This paper
sciP1.18_AGGTTGCC: AATGATACGGCGA CCACCGAGATCTACACAGGTTGCCCGG AGCTTTGCTAACGGTCG	IDT	This paper
sciP1.19_CCTTATGT: AATGATACGGCGA CCACCGAGATCTACACCCTTATGTCGGA GCTTTGCTAACGGTCG	IDT	This paper
sciP1.20_CAGCAACG: AATGATACGGCGA CCACCGAGATCTACACCAGCAACGCGG AGCTTTGCTAACGGTCG	IDT	This paper
sciP1.21_GGTTCAAT: AATGATACGGCGA CCACCGAGATCTACACGGTTCAATCGGA GCTTTGCTAACGGTCG	IDT	This paper
sciP1.22_ACATTCGT: AATGATACGGCGA CCACCGAGATCTACACACATTCGTCGGA GCTTTGCTAACGGTCG	IDT	This paper
sciP1.23_GATTCCCA: AATGATACGGCGAC CACCGAGATCTACACGATTCCCACGGAG CTTTGCTAACGGTCG	IDT	This paper
sciP1.24_CGGACTGC: AATGATACGGCGA CCACCGAGATCTACACCGGACTGCCGG AGCTTTGCTAACGGTCG	IDT	This paper
sciP1.25_AGCCGTTC: AATGATACGGCGAC CACCGAGATCTACACAGCCGTTCCGGAG CTTTGCTAACGGTCG	IDT	This paper
sciP1.26_ATTGGGTC: AATGATACGGCGAC CACCGAGATCTACACATTGGGTCCGGAG CTTTGCTAACGGTCG	IDT	This paper
sciP1.27_TGCATACT: AATGATACGGCGAC CACCGAGATCTACACTGCATACTCGGAGC TTTGCTAACGGTCG	IDT	This paper
sciP1.28_GGGCTTGG: AATGATACGGCGA CCACCGAGATCTACACGGGCTTGGCGG AGCTTTGCTAACGGTCG	IDT	This paper
sciP1.29_GACGTGGC: AATGATACGGCGA CCACCGAGATCTACACGACGTGGCCGGA GCTTTGCTAACGGTCG	IDT	This paper
sciP1.30_GCAAATTT: AATGATACGGCGAC CACCGAGATCTACACGCAAATTTCGGAG CTTTGCTAACGGTCG	IDT	This paper
sciP1.31_GCAGCCTC: AATGATACGGCGA CCACCGAGATCTACACGCAGCCTCCGGA GCTTTGCTAACGGTCG	IDT	This paper
sciP1.32_TCCGAGTT: AATGATACGGCGA CCACCGAGATCTACACTCCGAGTTCGGA GCTTTGCTAACGGTCG	IDT	This paper
sciP1.33_GCATTAAG: AATGATACGGCGAC CACCGAGATCTACACGCATTAAGCGGAG CTTTGCTAACGGTCG	IDT	This paper
sciP1.34_ACGATAAC: AATGATACGGCGA CCACCGAGATCTACACACGATAACCGGA GCTTTGCTAACGGTCG	IDT	This paper
sciP1.35_CCTGCGGG: AATGATACGGCGA CCACCGAGATCTACACCCTGCGGGCGGA GCTTTGCTAACGGTCG	IDT	This paper
sciP1.36_TGATTGTT: AATGATACGGCGAC CACCGAGATCTACACTGATTGTTCGGAGC TTTGCTAACGGTCG	IDT	This paper
sciP2.01_TAAGGCGA: CAAGCAGAAGACG GCATACGAGATTAAGGCGACTTACGGAT GTTGCACCAGC	IDT	This paper
sciP2.02_CGTACTAG: CAAGCAGAAGACGG CATACGAGATCGTACTAGCTTACGGATGT TGCACCAGC	IDT	This paper
sciP2.03_AGGCAGAA: CAAGCAGAAGACG GCATACGAGATAGGCAGAACTTACGGAT GTTGCACCAGC	IDT	This paper
sciP2.04_TCCTGAGC: CAAGCAGAAGACG GCATACGAGATTCCTGAGCCTTACGGATG TTGCACCAGC	IDT	This paper
sciP2.05_GGACTCCT: CAAGCAGAAGACG GCATACGAGATGGACTCCTCTTACGGAT GTTGCACCAGC	IDT	This paper
sciP2.06_TAGGCATG: CAAGCAGAAGAC GGCATACGAGATTAGGCATGCTTACGG ATGTTGCACCAGC	IDT	This paper
sciP2.07_CTCTCTAC: CAAGCAGAAGACG GCATACGAGATCTCTCTACCTTACGGAT GTTGCACCAGC	IDT	This paper
sciP2.08_CAGAGAGG: CAAGCAGAAGA CGGCATACGAGATCAGAGAGGCTTAC GGATGTTGCACCAGC	IDT	This paper
sciP2.09_GCTACGCT: CAAGCAGAAGACG GCATACGAGATGCTACGCTCTTACGGAT GTTGCACCAGC	IDT	This paper
sciP2.10_CGAGGCTG: CAAGCAGAAGAC GGCATACGAGATCGAGGCTGCTTACGG ATGTTGCACCAGC	IDT	This paper
sciP2.11_AAGAGGCA: CAAGCAGAAGAC GGCATACGAGATAAGAGGCACTTACGG ATGTTGCACCAGC	IDT	This paper
sciP2.12_GTAGAGGA: CAAGCAGAAGACG GCATACGAGATGTAGAGGACTTACGGAT GTTGCACCAGC	IDT	This paper
sciP2.13_TGGATCTG: CAAGCAGAAGACG GCATACGAGATTGGATCTGCTTACGGAT GTTGCACCAGC	IDT	This paper
sciP2.14_CCGTTTGT: CAAGCAGAAGACG GCATACGAGATCCGTTTGTCTTACGGAT GTTGCACCAGC	IDT	This paper
sciP2.15_TGCTGGGT: CAAGCAGAAGACG GCATACGAGATTGCTGGGTCTTACGGAT GTTGCACCAGC	IDT	This paper
sciP2.16_AGGTTGGG: CAAGCAGAAGACG GCATACGAGATAGGTTGGGCTTACGGAT GTTGCACCAGC	IDT	This paper
sciP2.17_GTGTGGTG: CAAGCAGAAGACG GCATACGAGATGTGTGGTGCTTACGGAT GTTGCACCAGC	IDT	This paper
sciP2.18_TGGGTTTC: CAAGCAGAAGACGGCAT ACGAGATTGGGTTTCCTTACGGATGTTGCAC CAGC	IDT	This paper
sciP2.19_TGGTCACA: CAAGCAGAAGACG GCATACGAGATTGGTCACACTTACGGAT GTTGCACCAGC	IDT	This paper
sciP2.20_TTGACCCT: CAAGCAGAAGACG GCATACGAGATTTGACCCTCTTACGGAT GTTGCACCAGC	IDT	This paper
sciP2.21_CGCGGACA: CAAGCAGAAGACG GCATACGAGATCGCGGACACTTACGGAT GTTGCACCAGC	IDT	This paper
sciP2.22_TTCCATAT: CAAGCAGAAGACGG CATACGAGATTTCCATATCTTACGGATGTT GCACCAGC	IDT	This paper
sciP2.23_AATTCGTT: CAAGCAGAAGACGG CATACGAGATAATTCGTTCTTACGGATGTT GCACCAGC	IDT	This paper
sciP2.24_GGCGTCGA: CAAGCAGAAGACG GCATACGAGATGGCGTCGACTTACGGAT GTTGCACCAGC	IDT	This paper
MEComp: /5Phos/C∗T∗G∗T∗C∗T∗C∗T∗T∗A∗T∗A∗C∗A∗/3ddC/	IDT	This paper

**Recombinant DNA**		

pTXB1-Tn5	Addgene	60240

**Software and algorithms**		

Bowtie 2 (v 2.3.3.1)	Langmead and Salzberg, 2012	http://bowtie-bio.sourceforge.net/bowtie2/index.shtml
SAMtools (v 1.9)	Li et al., 2009	http://samtools.sourceforge.net
Picard toolkit (2.14.1-SNAPSHOT)	N/A	http://broadinstitute.github.io/picard
chromVAR R package (v 0.2.0)	Schep et al., 2017	https://github.com/GreenleafLab/chromVAR
Trim Galore (v 0.6.6)	N/A	https://github.com/FelixKrueger/TrimGalore
MACS2 (v 2.1.2)	Zhang et al., 2008	https://github.com/taoliu/MACS/

**Other**		

Eppendorf ThermoMixer C	Eppendorf	Cat# 5382000023
BioRad T100 Thermal Cycler	Bio-Rad	Cat# 1861096
Bioruptor Plus	Diagenode	Cat# B01020001
Pipet-Lite Multi Pipette L8-20XLS+	Rainin	Cat# 17013803
Pipet-Lite Multi Pipette L8-200XLS+	Rainin	Cat# 17013805
Hemocytometer	Thermo Scientific Fisher	Cat# 02-671-6
Flowmi 40-micron cell strainers for 1000 microliter pipette tips	SP Bel-Art	Cat# H13680-0040
FlashGelTM Dock	Lonza Bio	Cat# 57025
ViiA 7 Real-Time PCR System	Applied Biosystems	Cat# 4453545
Falcon 14-mL round bottom polypropylene round bottom tubes	VWR	Cat# 60819-761/352059
SmartSpec Plus Spectrophotometer	Bio-Rad	N/A
BD FACSAria III Sorter	BD	N/A

## Materials and equipment

**Table:** Sci-oligo mix

Reagent	Final concentration	Amount
100 μM sciAD1.x or sciAD2.x	50 μM	13 μL
100 μM ME-comp oligo /5Phos/C∗T∗G∗T∗C∗T∗C∗T∗T∗A∗T∗A∗C∗A∗/3ddC/	50 μM	13 μL
1 M Tris pH 8.0	10 mM	0.26 μL
5 M NaCl	50 mM	0.26 μL
**Total**	**n/a**	**26.52 μL**

***Note:*** Sci-oligo mixes can be stored at −20°C or −80°C long term.

**Table:** HEGX Buffer

Reagent	Final concentration	Amount
1 M HEPES-KOH, pH 7.2	20 mM	2 mL
5 M NaCl	0.8 M	16 mL
0.5 M EDTA	1 mM	200 μL
Glycerol	10%	10 mL
10% Triton X-100	0.2%	2 mL
ddH_2_O	n/a	69.8 mL
**Total**	**n/a**	**100 mL**

***Note:*** Add KOH to HEPES buffer if pH needs to be adjusted. Prepare fresh HEGX Buffer for each experiment.

**Table:** Tn5 Dialysis Buffer

Reagent	Final concentration	Amount
1 M HEPES-KOH, pH 7.2	100 mM	100 mL
5 M NaCl	0.2 M	40 mL
0.5 M EDTA	0.2 mM	400 μL
1 M DTT	2 mM	2 mL
10% Triton X-100	0.2%	2 mL
Glycerol	20%	200 mL
ddH_2_O	n/a	655.6 mL
**Total**	**n/a**	**1000 mL**

***Note:*** Prepare fresh Tn5 Dialysis Buffer for each experiment.

**Table:** Dilution Buffer

Reagent	Final concentration	Amount
100% glycerol	50%	50 μL
1 M Tris pH 7.5	50 mM	5 μL
5 M NaCl	100 mM	2 μL
5 mM EDTA	0.1 mM	2 μL
100 mM DTT	1 mM	1 μL
10% NP-40	0.1%	1 μL
nuclease free H_2_O	n/a	39 μL
**Total**	**n/a**	**100 μL**

***Note:*** Dilution buffer can be stored at −20°C for several months.

**Table:** FACS Buffer

Reagent	Final concentration	Amount
1× PBS	n/a	49.55 mL
0.5 M EDTA	2 mM	200 μL
250 μL heat-inactivated FBS	n/a	250 μL
**Total**	**n/a**	**50 mL**

***Note:*** FACS Buffer can be stored at 4°C for 2 weeks.

**Table:** Transposition Buffer

Reagent	Final concentration	Amount
0.2 M Tris-acetate	41.25 mM	297 μL
5 M K-acetate	82.5 mM	23.76 μL
1 M Mg-acetate	12.5 mM	18 μL
100% DMF (water free)	20%	288 μL
10% NP-40	0.125%	18 μL
Protease Inhibitor Cocktail	0.5%	7.2 μL
nuclease free H_2_O	n/a	788.04 μL
**Total**	**n/a**	**1.44 mL**

***Note:*** Prepare fresh transposition buffer for each experiment.

**Table:** Nuclei Isolation Buffer

Reagent	Final concentration	Amount
1 M Tris HCl pH 7.5	10 mM	0.5 mL
5 M NaCl	10 mM	0.1 mL
1 M MgCl_2_	3 mM	0.15 mL
10% NP40 (IGEPAL CA-630)	0.1%	0.5 mL
nuclease free H_2_0	n/a	48.75 mL
**Total**	**n/a**	**50 mL**

***Note:*** Nuclei Isolation Buffer can be stored at 4°C for 2 weeks.

**Table:** 2× Reverse crosslinking buffer (RCB)

Reagent	Final concentration	Amount
1 M Tris pH 8.0	100 mM	1 mL
5 M NaCl	100 mM	0.2 mL
20% SDS	0.40%	0.2 mL
nuclease free H_2_O	n/a	8.58 mL
**Total**	**n/a**	**10 mL**

***Note:*** 2× Reverse crosslinking buffer can be stored at room temperature (25°C) for several months.

**Table:** qPCR master mix (1×)

Reagent	Final concentration	Amount
2× NEBNext	n/a	5 μL
5 μM sciP1.01	0.5 μM	0.20 μL
5 μM sciP2.01	0.5 μM	0.20 μL
10× SYBR	0.6×	0.90 μL
nuclease free H_2_0	n/a	3.70 μL
**Total**	**n/a**	**10 μL**

***Note:*** Prepare fresh qPCR master mix for each experiment.

***Note:*** Any sciP1.X and sciP2.X primer can be used in this mix. The fragments will not be sequenced so the primers do not need to match the samples’ barcodes.

***Note:*** SYBR mix is purchased as a 10,000× concentrate in DMSO. This stock should be further diluted to a 10× stock using water. Storage in a foil-covered 1.5 mL Eppendorf microcentrifuge tube at −20°C is advised. Make sure that qPCR master mix is well mixed before use.

***Note:*** Scale up master mix depending on sample number.

**Table:** KAPA dilution buffer

Reagent	Final concentration	Amount
Tris-HCl pH 8.0	10 mM	0.5 mL
Tween20	0.05%	25 μL
nuclease free H_2_0	n/a	49.5 mL
**Total**	**n/a**	**50 mL**

***Note:*** KAPA dilution buffer can be stored at room temperature (25°C) for several months.

**CRITICAL:**

SYBR (Thermo Fisher Scientific, Cat# S7563) is a combustible liquid. Handle with appropriate safety precautions.

16% Methanol free formaldehyde (Thermo Fisher Scientific, Cat# 28908) is harmful if swallowed, inhaled, or in contact with skin, causing skin and eye irritation. Handle with appropriate safety precautions.

SDS (Bio-Rad, Cat# 161-0302) is flammable, harmful if swallowed, causes skin and eye irritation, and may cause respiratory irritation. Handle with appropriate safety precautions.

NP-40 Surfact-Amps Detergent Solution (Thermo Scientific Nalgene, Cat# 28324) causes skin and eye irritation. Handle with appropriate safety precautions.

DMF (Thermo Fisher Scientific, Cat# 20673) is flammable, harmful in contact with skin or if inhaled, causes skin and eye irritation, and may damage fertility. Handle with appropriate safety precautions.

DTT (Thermo Fisher Scientific, Cat# R0861) is harmful if swallowed, causes skin irritation, and causes serious eye irritation. Handle with appropriate safety precautions.

***Alternatives:***

All standard reagents in the protocol can be replaced by comparable reagents from other manufacturers.

This protocol requires preparation and optimization of lab purified Tn5. Unloaded Tn5 is commercially available but may not be as cost effective for this protocol.

This protocol uses Lonza FlashGels^TM^ to image the ATAC fragment sizes. Samples can also be run on standard agarose gels.

This protocol uses a BD FACSAria III sorter, but any comparable cell sorter can be used.

This protocol uses a ViiA7 Real-Time PCR system, but any comparable qPCR machine can be used.

## Step-by-step method details

This protocol includes a number of adaptions and optimization from prior methodologies (Chen et al., 2018, Cusanovich et al., 2015, Lake et al., 2018, Pliner et al., 2018, Preissl et al., 2018).

### Sample preparation

**Figure 2:**
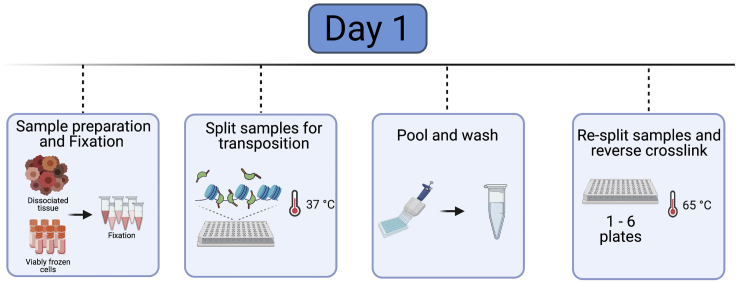
Overview of Day 1 of the protocol before an overnight stopping point Day 1 of the protocol includes sample preparation and fixation, transposition in a 96 well plate, sample pooling, re-splitting the samples and reverse crosslinking. Reverse crosslinking occurs overnight and the protocol is resumed at Day 2. Created with biorender.com.

Preparation of samples will depend on sample collection methodology (Figure 2, Day 1 overview). For cell lines, we recommend detachment of cells with trypsin or accutase and then proceed to fixation and transposition. Fresh cells should be isolated or viably frozen cells should be thawed immediately and washed 1–2 times with 1× PBS before the start of the protocol. This protocol describes the specific steps for using fresh murine lung tumor samples (LaFave et al., 2020). Lung tumors are isolated from Kras^G12D/+^; Trp53^-/-^; Rosa26^tom/+^ mice and dissociated cells are sorted for tdTomato expression to isolate tumor cells for sciATAC-seq. Tumors are induced at 6–8 weeks of age and late stage tumors are collected at 25 weeks post-tumor induction (LaFave et al., 2020). Cell preparations can be used fresh or viably frozen in media (DMEM + 10% FCS) and 10% DMSO; however, we recommend performing the sort of the day of the experiment on viably frozen tumor cells.

**Timing: [Day 1]**

**Figure 3:**
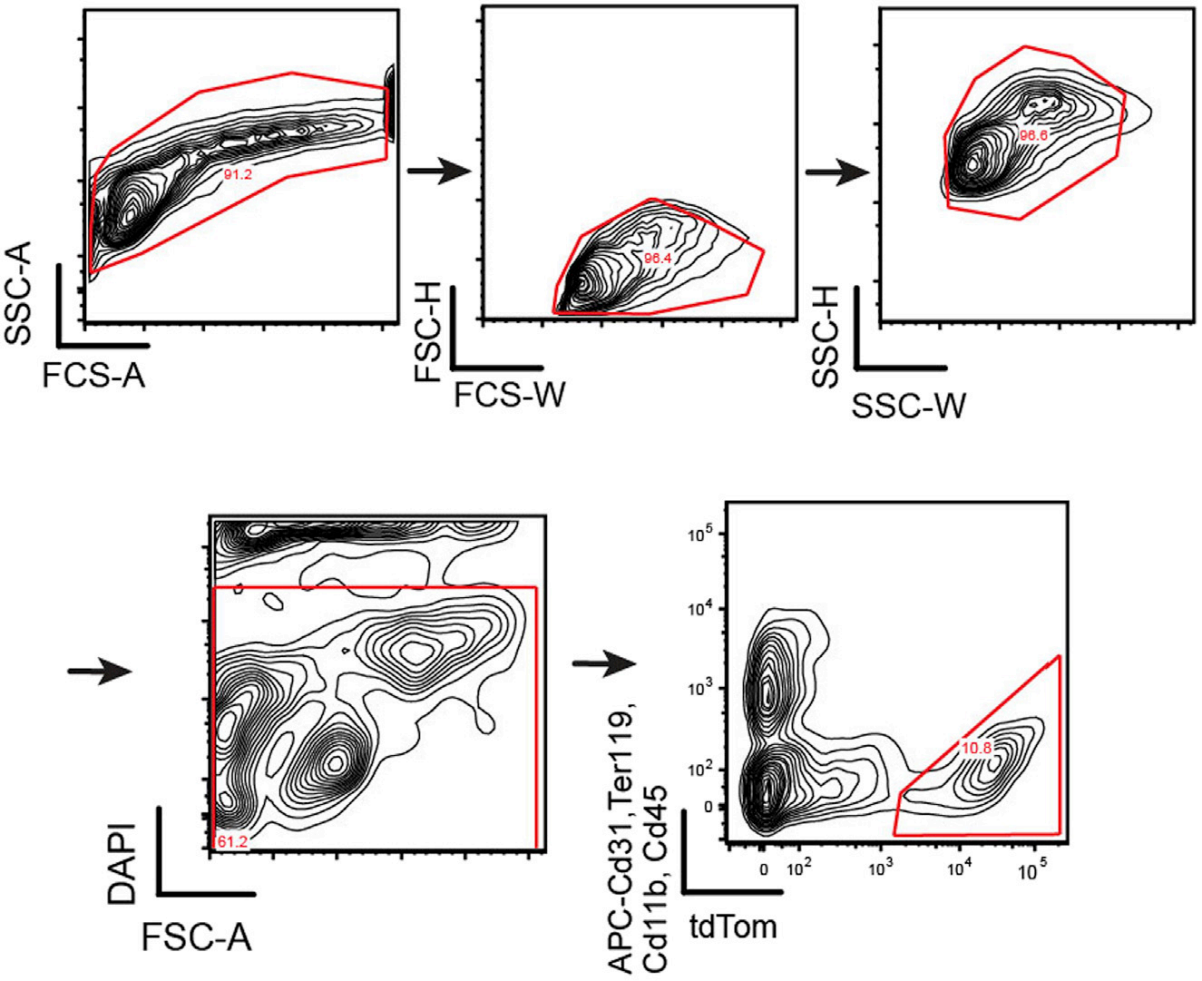
Gating strategy for lung adenocarcinoma cells FACS-depletion strategy is shown (TER119-APC, CD31-APC, CD11b-APC, and CD19-APC) and DAPI for a live-dead stain. tdTom^+^ positive populations (APC negative, DAPI positive) are collected for fixation and transposition. Figure reprinted with permission from LaFave et al., 2020.

1. Collection of lung adenocarcinoma cells
  **Timing: [4 hrs]**
  a. Euthanize mouse using an isoflurane chamber.
  b. Dissect whole tumor-burdened lung or individual tumors and place in 1.5 mL Eppendorf microcentrifuge tubes containing 100 μL Lung Digestion Buffer. Buffer is based on manufacturer’s instructions (https://www.miltenyibiotec.com/US-en/products/lung-dissociation-kit-mouse.html#gref).
  c. Thinly mince tissues using scissors and then add an additional 0.5 mL Lung Digestion Buffer to tube.
  d. Incubate at 37°C with rotation for 30 min.
  e. Grind sample over a 100 μM filter into a 50 mL conical tube with pipette tip.
  f. Pipette 5 mL S-MEM over the filter to wash remaining sample into the tube.
  g. Pellet cells by centrifugation at 300 × *g* for 5 min.
    ***Note:*** Centrifugation speed and time may be adjusted according to sample type.
  h. Remove supernatant and resuspend pellet in 1 mL ACK lysis buffer to lyse red blood cells. Incubate on the bench at 25°C for 2 min.
  i. Quench reaction by adding 10 mL DMEM and pellet by centrifugation at 300 × *g* for 5 min.
  j. Resuspend pellet in 500 μL of FACS Buffer. Stain samples with APC-conjugated CD45 (BD, 559864), CD11b (eBioscience, 15-0112-82), CD31 (BioLegend, 102510), and Ter119 (BD, 557909) at a concentration of 1:1000, and DAPI (stock concentration 5 mg/mL; stain at a concentration of 1:10000).
    ***Note:*** Use of a dead cell removal kit (e.g., Miltenyi cat no. 103-090-101) is recommended.
  k. Sort for APC^neg^ (immune depleted), tdTomato^pos^, and DAPI^neg^ into FACS buffer using a FACS Aria (example gating scheme, Figure 3). Proceed to fixation and transposition.

### Fixation and transposition

Permeabilized cells are prepared and uniquely barcoded Tn5 transposases label open regions of chromatin.

**Timing: [Day 1]**

2. Assemble Tn5
  **Timing: [45 min]**
  a. Prepare Dilution Buffer, then cool on ice for 5 min. The dilution buffer can be stored at −20°C for several months.
    **CRITICAL:** Assemble Tn5 immediately before fixation and transposition steps.
  b. Prepare 1× Tn5 by diluting homemade Tn5 in Dilution Buffer to a final volume of 96 μL.
  c. Mix diluted Tn5 1:1 with pre-annealed adapters/glycerol mix by combining 4 μL diluted Tn5 with 4 μL each pre annealed adapter (20 adapters in total).
    ***Note:*** For 96 transposition reactions, 8 sciAD1.X and 12 sciAD2.X distinct barcodes are used, see Table 4 for barcode combinations.
    **Table 4:** Primer plate map for assembled oligos

sciAD1.3 + sciAD2.1	sciAD1.3 + sciAD2.3	sciAD1.3 + sciAD2.4	sciAD1.3 + sciAD2.6	sciAD1.3 + sciAD2.7	sciAD1.3 + sciAD2.8	sciAD.3 + sciAD2.11	sciAD1.3 + sciAD2.12	sciAD1.3 + sciAD2.13	sciAD1.3 + sciAD2.14	sciAD1.3 + sciAD2.15	sciAD1.3 + sciAD2.17
sciAD1.4 + sciAd2.1	sciAD1.4 + sciAD2.3	sciAD1.4 + sciAD2.4	sciAD1.4 + sciAD2.6	sciAD1.4 + sciAD2.7	sciAD1.4 + sciAD2.8	sciAD1.4 + sciAD2.11	sciAD1.4 + sciAD2.12	sciAdD.4 + sciAD2.13	sciAD1.4 + sciAD2.14	sciAD1.4 + sciAD2.15	sciAD1.4 + sciADd2.17
sciAD1.5 + sciAD2.1	sciAD1.5 + sciAD2.3	sciAD1.5 + sciAD2.4	sciAD1.5 + sciAD2.6	sciAD1.5 + sciAD2.7	sciAD1.5 + sciAD2.8	sciAD1. 5 + sciAD2.11	sciAD1.5 + sciAD2.12	sciAD1.5 + sciAD2.13	sciAD1.5 + sciAD2.14	sciAD1.5 + sciAD2.15	sciAD1.5 + sciAD2.17
sciAD1.6 + sciAD2.1	sciAD1.6 + sciAD2.3	sciAD1.6 + sciAD2.4	sciAD1.6 + sciAD2.6	sciAD1.6 + sciAD2.7	sciAD1.6 + sciAD2.8	sciAD1.6 + sciaD2.11	sciAD1.6 + sciAD2.12	sciAD1.6 + sciAD2.13	sciAD1.6 + sciAD2.14	sciAD1.6 + sciAD2.15	sciAD1.6 + sciAD2.17
sciAD1.7 + sciAD2.1	sciAD1.7 + sciAD2.3	sciAD1.7 + sciAD2.4	sciAD1.7 + sciAD2.6	sciAD1.7 + sciAD2.7	sciAD1.7 + sciAD2.8	sciAD1.7 + sciAD2.11	sciAD1.7 + sciAD2.12	sciAD1.7 + sciAD2.13	sciAD1.7 + sciAD2.14	sciAD1.7 + sciAD2.15	sciAD1.7 + sciAD2.17
sciAD1.9 + sciAD2.1	sciAD1.9 + sciAD2.3	sciAD1.9 + sciAD2.4	sciAD1.9 + sciAD2.6	sciAD1.9 + sciAD2.7	sciAD1.9 + sciAD2.8	sciAD1.9 + sciAD2.11	sciAD1.9 + sciAD2.12	sciAD1.9 + sciAD2.13	sciAD1.9 + sciAD2.14	sciAD1.9 + sciAD2.15	sciAD1.9 + sciAD2.17
sciAD1.10 + sciAD2.1	sciAD1.10 + sciAD2.3	sciAD1.10 + sciAD2.4	sciAD1.10 + sciAD2.6	sciAD1.10 + sciAD2.7	sciAD1.10 + sciAD2.8	sciAD1.10 + sciAD2.11	sciAD1.10 + sciA.D212	sciAD1.10 + sciAD2.13	sciAD1.10 + sciAD2.14	sciAD1.10 + sciAD2.15	sciAD1.10 + sciAD2.17
sciAD1.11 + sciAD2.1	sciAD1.11 + sci AD2.3	sciAD1.11 + sci AD2.4	sciAD1.11 + sciAD2.6	sciAD1.11 + sciAD2.7	sciAd1.11 + sciAD2.8	sciAD1.11 + sciA2D.11	sciAD1.11 + sciAD2.12	sciAD1.11 + sciAD2.13	sciAD1.11 + sciAD2.14	sciAD1.11 + sci sciAD2.15	sciAD1.11 + sciAD2.17
  d. Incubate on the bench at 25°C for 30 min.
  e. After 30 min, proceed to fixation and transposition steps but keep on ice.

3. Fixation
  **Timing: [1 hr]**
  a. Pellet cells by centrifugation at 300 × *g* for 3 min.
    **CRITICAL:** Always coat tubes with 7.5% BSA before spinning to improve cell recovery. Add BSA to coat the entire tube and remove prior to cell fixation.
    **CRITICAL:** Always use a swing bucket rotor for centrifugation instead of a tabletop centrifuge.
    ***Note:*** Centrifugation time and speed may need to be adjusted for sample type based on prior experience.
  b. Resuspend pellets in 100 μL cold 1× PBS.
  c. Count cells by mixing 5 μL of each sample to 5 μL of trypan blue using a hemocytometer.
    ***Note:*** Visually check samples for clumping and debris which may cause issues in future steps (Troubleshooting 2).
  d. Dilute each sample to 100K cells in a final volume of 100 μL.
    ***Note:*** If cell concentration cannot be achieved, resuspend the entire cell sample in 100 μL. Variations to cell number must be accounted for at the PCR barcoding step by maintaining an appropriate cell density per reaction.
  e. Pellet cells by centrifugation at 300 × *g* for 3 min.
  f. Dilute 16% formaldehyde to 1.6% with H_2_O.
  g. Fix cells by adding 6.7 μL 1.6% formaldehyde (final concentration 0.1%). Mix samples by pipetting (Troubleshooting 3).
    ***Note:*** Fixation conditions have been optimized in specific human and mouse cells lines and have been successful in several tissues including lung and brain. Fixation percentage can be tuned from 0.1%–0.5% depending on cell fragility.
    ***Note:*** Omitting the fixation step may enable experiments with limited cell number, but will increase the level of crosstalk between single cells due to excess debris in the sample prep (LaFave et al., 2020).
  h. Incubate on the bench at 25°C for 5 min.
  i. Prepare a master mix of 5.6 μL 2.5M glycine, 5.0 μL 1 M pH 8.0 Tris, and 1.3 μL 7.5% BSA for each 100 μL fixed sample. Stop the fixation by adding 11.9 uL master mix to each individual sample.
  j. Incubate on ice for 10 min.
  k. Gently wash the cells with 0.5 mL of 1× PBS by pipetting against the side of the tube without resuspending the pellet. Spin at 500 × *g* for 3 min.
  l. Repeat step k.
  m. Resuspend pellets in 1 μL 1× PBS for each transposition reaction as necessary for pre-determined number of reactions.

***Note:*** Use approximately 1K cells for each transposition reaction. Adjust the number of reactions per sample depending on desired number of reactions (e.g., if performing 12 transposition reactions per sample, resuspend 12K cells in 12 μL 1× PBS). In total, samples should be split across the 96-well plate (e.g., an example experiment may be 8 samples and 12 transposition reactions per sample to complete the plate).

***Note:*** Multiplexed barcoding allows for transposition of different samples/conditions in the same experiment. Samples should remain separated at this step for labeling by transposition. Based on what percentage of cells needed from each sample/condition, the researcher will need to determine the number of 96 wells that correspond to each sample. The researcher should keep track of the sample in each well for demultiplexing purposes.

4. Transposition
  **Timing: [1.5 h]**
  a. Prepare Transposition Buffer and Nuclei Isolation Buffer.
  b. In a 96 well plate, combine 7 μL of the transposition buffer and 1 μL fixed cells in each well.
  c. Incubate on the bench at 25°C for 10 min.
  d. Dilute the assembled Tn5 1:1 by adding 8 μL Transposition Buffer to 8 μl assembled Tn5. Add 1 μL diluted Tn5 containing sciAD1.X oligo and 1 μL diluted Tn5 containing sciAD2.X oligo to each well based on plate map (Table 4).
  e. Shake at 300 rpm for 30 min at 37°C using the Eppendorf ThermoMixer C.
  f. Quench the reaction by adding 1 μL of 0.5M EDTA to each well. Mix well by pipetting.
  g. Shake at 300 rpm for 15 min at 37°C.

### Combine transposition reactions and prepare for PCR barcoding

Transposed samples are pooled together and re-aliquoted into individual wells to add another level of barcoding at PCR amplification.

5. Pool transposition reactions
  **Timing: [30 min]**
  a. Pool all reactions from each well of the 96 well plate (approximately 1 mL in total) into a BSA-coated 1.5 mL Eppendorf microcentrifuge tube. Add 38.4 μL 1M MgCl_2_ to quench EDTA and mix by pipetting.
  b. Pellet cells by centrifugation at 500 × *g* for 2 min.
  c. Remove supernatant and wash cells with 1 mL Nuclei Isolation Buffer before centrifugation at 500 × *g* for 2 min.
  d. Resuspend pellet with 0.5 mL Nuclei Isolation Buffer and pass the entire volume through a 40 μm Flowmi cell strainer for 1000 μL pipette tips into a new Eppendorf tube.
  e. Count cells using a hemocytometer and dilute transposed cells to a concentration of 13.3 cells/μL in Nuclei Isolation Buffer.

***Note:*** Final volume is dependent on the number of 96 well plates used for PCR amplification.

6. Reverse crosslinking
  **Timing: [18 h]**
  a. Prepare 2× Reverse Crosslinking Buffer (RCB).
  b. Add 2 μL of 20 mg/mL proteinase K per 1 mL of RCB.
    ***Note:*** 1 mL of RCB is required for each PCR plate. To increase recovery of cells, the protocol can be scaled up to 9 individual 96 well PCR plates.
  c. Create master mixes for each sciP1.x primer and each sciP2.x primer to aliquot in a 96 well plate.
    ***Note:*** An example of how to aliquot primers across the plate can be found in Table 5.
    **Table 5:** Example plate map for PCR amplification

sciP1.01 + sciP2.01	sciP1.02 + sciP2.01	sciP1.03 + sciP2.01	sciP1.04 + sciP2.01	sciP1.05 + sciP2.01	sciP1.06 + sciP2.01	sciP1.07 + sciP2.01	sciP1.08 + sciP2.01	sciP1.09 + sciP2.01	sciP1.10 + sciP2.01	sciP1.11 + sciP2.01	sciP1.12 + sciP2.01
sciP1.01 + sciP2.02	sciP1.02 + sciP2.02	sciP1.03 + sciP2.02	sciP1.04 + sciP2.02	sciP1.05 + sciP2.02	sciP1.06 + sciP2.02	sciP1.07 + sciP2.02	sciP1.08 + sciP2.02	sciP1.09 + sciP2.02	sciP1.10 + sciP2.02	sciP1.11 + sciP2.02	sciP1.12 + sciP2.02
sciP1.01 + sciP2.03	sciP1.02 + sciP2.03	sciP1.03 + sciP2.03	sciP1.04 + sciP2.03	sciP1.05 + sciP2.03	sciP1.06 + sciP2.03	sciP1.07 + sciP2.03	sciP1.08 + sciP2.03	sciP1.09 + sciP2.03	sciP1.10 + sciP2.03	sciP1.11 + sciP2.03	sciP1.12 + sciP2.03
sciP1.01 + sciP2.04	sciP1.02 + sciP2.04	sciP1.03 + sciP2.04	sciP1.04 + sciP2.04	sciP1.05 + sciP2.04	sciP1.06 + sciP2.04	sciP1.07 + sciP2.04	sciP1.08 + sciP2.04	sciP1.09 + sciP2.04	sciP1.10 + sciP2.04	sciP1.11 + sciP2.04	sciP1.12 + sciP2.04
sciP1.01 + sciP2.05	sciP1.02 + sciP2.05	sciP1.03 + sciP2.05	sciP1.04 + sciP2.05	sciP1.05 + sciP2.05	sciP1.06 + sciP2.05	sciP1.07 + sciP2.05	sciP1.08 + sciP2.05	sciP1.09 + sciP2.05	sciP1.10 + sciP2.05	sciP1.11 + sciP2.05	sciP1.12 + sciP2.05
sciP1.01 + sciP2.06	sciP1.02 + sciP2.06	sciP1.03 + sciP2.06	sciP1.04 + sciP2.06	sciP1.05 + sciP2.06	sciP1.06 + sciP2.06	sciP1.07 + sciP2.06	sciP1.08 + sciP2.06	sciP1.09 + sciP2.06	sciP1.10 + sciP2.06	sciP1.11 + sciP2.06	sciP1.12 + sciP2.06
sciP1.01 + sciP2.07	sciP1.02 + sciP2.07	sciP1.03 + sciP2.07	sciP1.04 + sciP2.07	sciP1.05 + sciP2.07	sciP1.06 + sciP2.07	sciP1.07 + sciP2.07	sciP1.08 + sciP2.07	sciP1.09 + sciP2.07	sciP1.10 + sciP2.07	sciP1.11 + sciP2.07	sciP1.12 + sciP2.07
sciP1.01 + sciP2.08	sciP1.02 + sciP2.08	sciP1.03 + sciP2.08	sciP1.04 + sciP2.08	sciP1.05 + sciP2.08	sciP1.06 + sciP2.08	sciP1.07 + sciP2.08	sciP1.08 + sciP2.08	sciP1.09 + sciP2.08	sciP1.10 + sciP2.08	sciP1.11 + sciP2.08	sciP1.12 + sciP2.08
  d. Prepare Master Mix 1 with the transposed cell sample (concentration 13.3 cells/μL determined above) and 10 μM sciP1.X for each row. To do this, add 15 μL of the transposed cells to 5 μL of each primer sciP1.X (12 total).
  e. Prepare Master Mix 2 with 10 μM sciP2.X and RCB with proteinase K for each column. To do this, add 50 μL RCB + proteinase K to 10 μL each primer sciP2.X (8 total).
    ***Note:*** Prepare Master Mix 1 and Master Mix 2 in PCR tubes to allow for multichannel pipetting when distributing across the 96 well plate.
  f. Distribute 2 μL of Master Mix 1 across the 96-well plate according to the example in Table 5.
  g. Distribute 3 μL of Master Mix 2 across the 96-well plate according to the example in Table 5.
    ***Note:*** Each well should have 2.5 μL RCB with proteinase K, 0.5 μL 10 μM sciP1.X, 0.5 μL 10 μM sciP2.X, and 1.5 μL transposed cells. Master Mix 2 is made in excess and leftover is expected.
    ***Note:*** In the nomenclature for PCR barcodes, sciP1.X and sciP2.X, X refers to the full repertoire of barcoding space.
  h. Incubate in a thermal cycler at 55°C for 1 h to 16 h.

**Pause point:** Shorter incubation times from 1–16 h can be used. We recommend 16 h to provide a convenient stopping point between Day 1 and Day 2 of the protocol.

### PCR amplification and quantification

**Figure 4:**
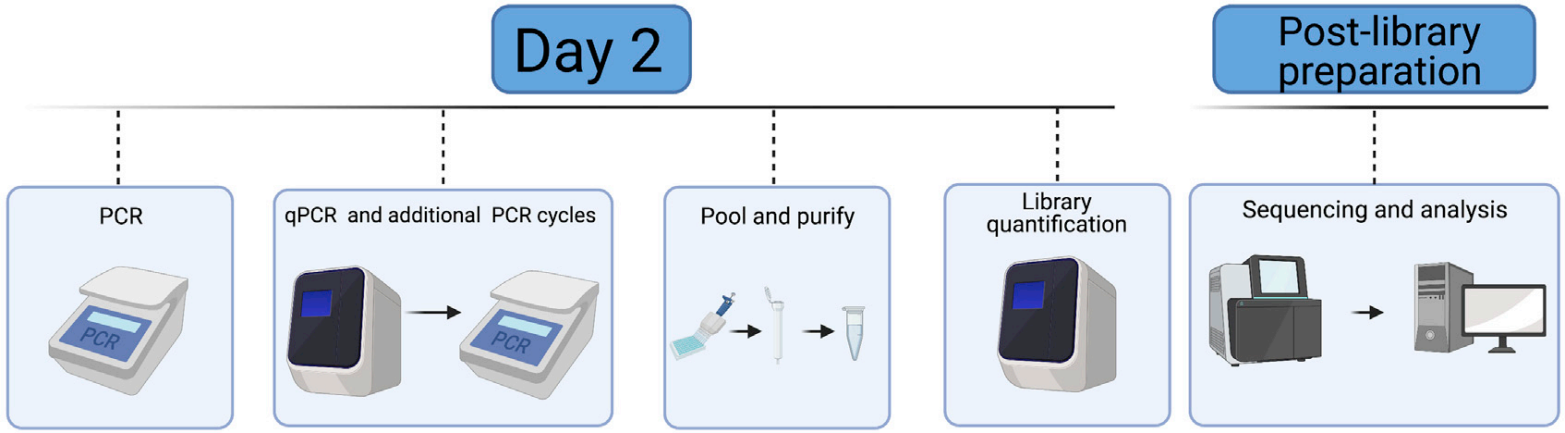
Overview of Day 2 of the protocol and downstream analysis Day 2 of the protocol requires PCR of split samples, qPCR to identify the number of additional PCR cycles required for minimal library amplification, pooling of libraries and library quantification. The libraries can then be sequenced and analyzed for downstream applications. Created with biorender.com

Addition of barcodes through PCR and quality check prior to sequencing (Figure 4, Day 2 overview).

**Timing: [Day 2]**

7. PCR Amplification
  **Timing: [45 min]**
  a. Remove PCR plate from thermal cycler and briefly spin down plate at 300 × *g* for 10 seconds.
  b. Quench reaction by adding 5 μL 10% Tween20 to each well. Mix well by pipetting using a multichannel pipette.
  c. Prepare PCR master mix by combining 1.25 mL 2× NEBNext PCR mix and 250 μL nuclease free H_2_O.
  d. Add 15 μL of the PCR mix solution to each well using a multichannel pipette. Pipette to mix and spin down plate.
  e. Perform PCR reaction as follows:

**Table:** 

PCR cycling conditions			
Steps	Temperature	Time	Cycles
Initial Extension	72°C	5 min	1
Initial Denaturation	98°C	5 min	1
Denaturation	98°C	10 s	5
Annealing	70°C	30 s	
Extension	72°C	1 min	
Hold	4°C

8. Quantitative PCR
  **Timing: [2 h]**
  a. After the initial 5 cycles of amplification, remove the plate from the thermal cycler and keep on ice for the duration of this step.
  b. For a quality check, randomly choose 4–8 wells to use for quantitative PCR (qPCR) reactions to determine the number of additional cycles needed for the entire plate. A negative control using water instead of DNA is advised.
  c. Prepare qPCR master mix. Scale up based on the number of wells chosen to test above.
  d. Add 9 μL qPCR master each well of qPCR plate. Add 1 μL sample to each well.
  e. Perform qPCR reaction to saturation as follows:

**Table:** 

PCR cycling conditions			
Steps	Temperature	Time	Cycles
Initial Denaturation	98°C	30 s	1
Denaturation	98°C	10 s	25
Annealing	70°C	30 s	
Extension	72°C	1 min	
Hold	4°C
  f. Determine the number of additional PCR cycles. To do this, the fluorescence signal is normalized to the range of 0–1. The ⅓ C_T_ is calculated as the qPCR cycle number to reach 0.33. The additional PCR cycle number is calculated by subtracting 5 from ⅓ C_T_ value calculated by qPCR (Additional cycle N = ⅓ C_T_ - 5) (Figure 5).

**Figure 5:**
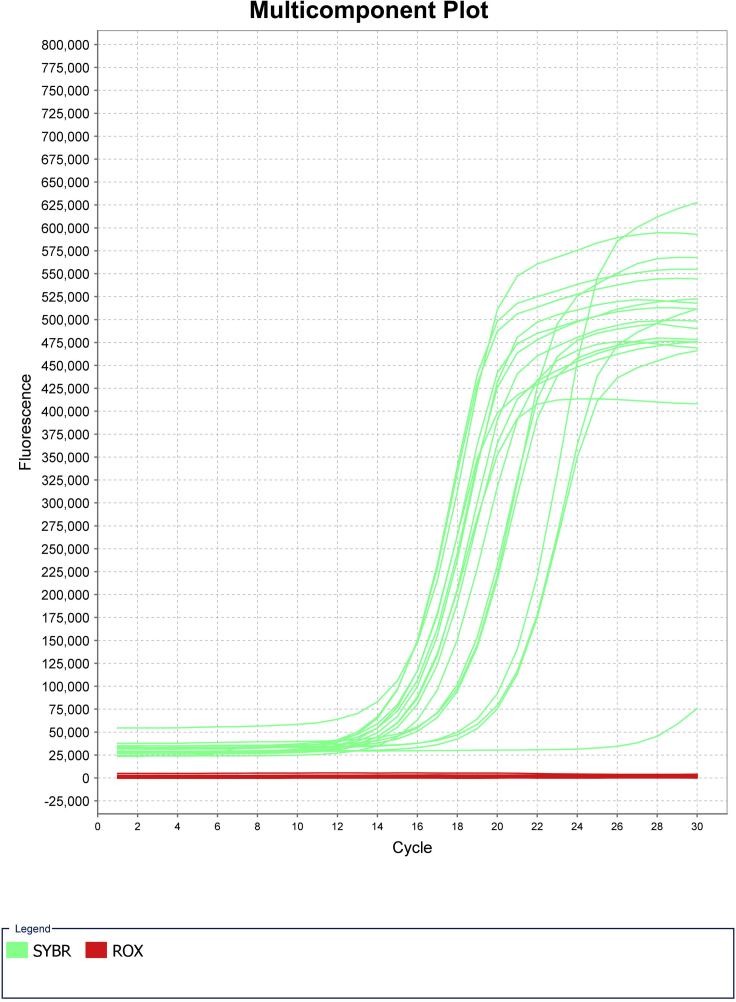
Example of qPCR results from successful sciATAC-seq run Negative controls shown by amplification curve with low C_T_ value and individual wells with higher C_T_ values. Some variability in C_T_ values across samples is possible, especially with primary samples.
  g. Perform additional cycles as follows:

**Table:** 

PCR cycling conditions			
Steps	Temperature	Time	Cycles
Initial Denaturation	98°C	30 s	1
Denaturation	98°C	10 s	N additional cycles
Annealing	70°C	30 s	
Extension	72°C	1 min	
Hold	4°C
  h. Run qPCR reactions on a Lonza FlashGel^TM^ DNA cassette 2.2% gel for 9 min at 250 Volts.
  i. After running, the gel is imaged in a Biorad Gel Doc XR or similar UV trans-illuminator.

***Note:*** Confirm that you see the expected fragmentation pattern following ATAC-seq (Figure 6). Failed experiment will appear as primer dimers and means that the experiment was not successful (Troubleshooting 4).

**Figure 7:**
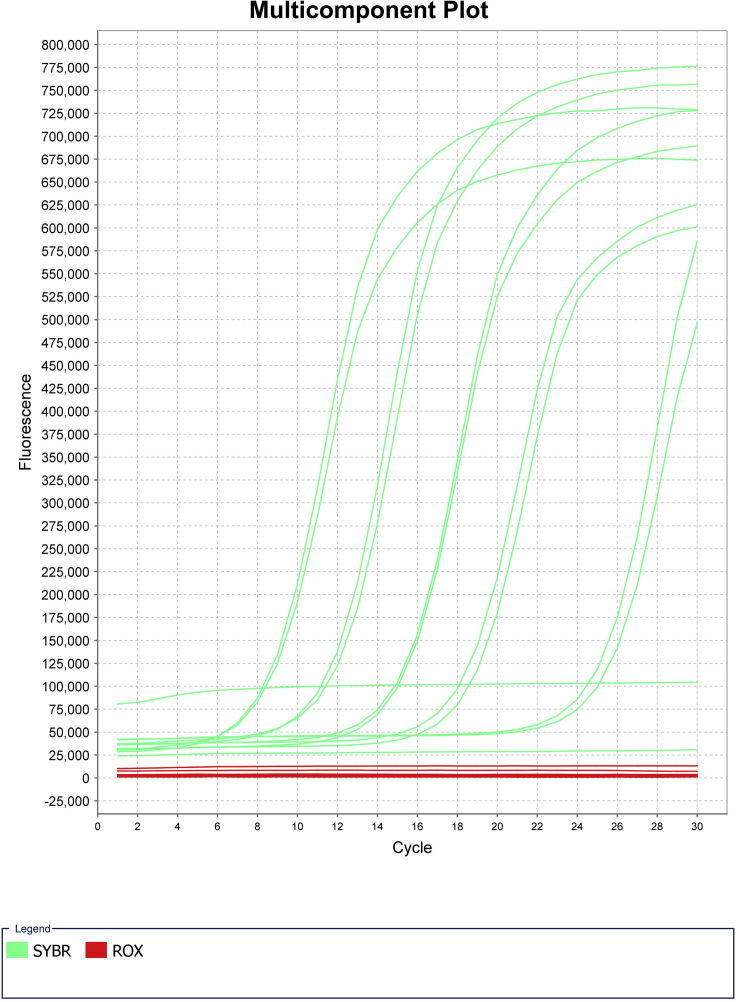
Example of KAPA quantification dilution series for library quantification
  e. Perform KAPA qPCR reaction as follows and perform library quantification based on KAPA kit instructions (https://rochesequencingstore.com/wp-content/uploads/2017/10/KAPA-Lib-Quant-ILMN_9.17-IfU_1.pdf) (Troubleshooting 5):

9. Quantification of minimally amplified libraries
  **Timing: [2 h]**
  a. Pool all the post-PCR samples into a 15 mL conical tube.
    ***Note:*** Pool samples from each plate into separate conical tubes.
  b. Purify DNA using Qiagen MinElute PCR purification column following manufacturer’s instructions (https://www.qiagen.com/us/resources/resourcedetail?id=c1276626-d0b2-4e95-b2c3-dc81803a198c&lang=en). Elute in 12 μL EB buffer.
    **Pause point:** Purified libraries can be stored at −20°C until quantification via qPCR.
  c. Dilute 1 μL sample 100,000× in KAPA dilution buffer. This can be accomplished by serially diluting the sample 100×, then 100×, then 10× for quantification of purified libraries using the KAPA quantification kit.
  d. Prepare qPCR reactions by adding 6 μL KAPA SYBR FAST qPCR Master Mix with added Primer Premix and 4 μL diluted sample or provided standards (Figure 7) to each well. Perform quantification reactions in triplicate.

**Table:** 

PCR cycling conditions			
Steps	Temperature	Time	Cycles
Initial Denaturation	95°C	5 min	1
Denaturation	95°C	30 s	35
Annealing	60°C	45 s	
Hold	4°C

**Figure 8:**
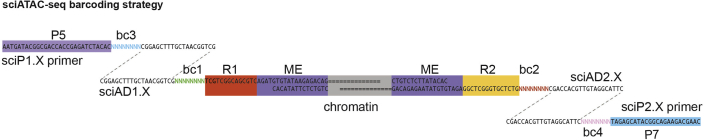
Overview of barcoding strategy The barcoding strategy has been updated to avoid sequencing with custom primers. sciAD1.X, sciAD2.X, sciP1.X, and sciP2.X ultimately assign four unique barcodes (bc1-4) per cell. Read 1 (R1) and Read 2 (R2) are flanked by the Tn5 mosaic end (ME) transposition recognition sequences.
  b. Convert base calls to fastq format using bcl2fastq.
  c. Trim sequencing reads to remove adapter sequences using trim_galore <filename(s)>. trim_galore automatically detects Nextera adapter sequences and trims low quality reads.
  d. Align reads to hg19 or mm10 genome using Bowtie2 (Langmead and Salzberg, 2012) using maximum fragment length set to 2 kb and all other default settings (bowtie2 -X2000 --rg-id).
  e. Demultiplex tolerating one mismatch base within barcodes.
  f. Remove mitochondrial and low-quality reads using SAMtools (Li et al., 2009) (samtools view -b -q 30 -f 0x2).
  g. Remove duplicate sequences with picard toolkit (http://broadinstitute.github.io/picard/)
  h. Call peaks from a single alignment (.bam) file using input peak calling with MACS v2.1.2 (MACS2). Use default options with the following flags set: -nomodel, -nolambda, -keep-dup all, -call-summits returning a list of single base pair peak summits with FDR *q* < 0.01.
    ***Note:*** If calling peaks across multiple experiments, aggregate each sample into one .bam file. Call peaks on each sample to obtain sample-specific peaks.
  i. Identify a list of significant, non-overlapping fixed width peak windows by padding peak summits with 150 base pairs at either side to generate 301 base pair window peak regions.
  j. Sort peaks in decreasing order of significance scores. Remove peak windows with the lower significance scores and keep the most significant peak. Repeat over an iterative process, to identify 301 base pair disjoin peak windows.
  k. Using the generated peak list above, determine the number of reads overlapping a given peak window for each unique cell barcode.
  l. Generate a peak X cell counts matrix which corresponds to ATAC reads in peaks for each cell profiled.

10. Next-generation sequencing and data processing
  a. Libraries can be sequenced on the Next-seq platform (Illumina) using a 150-cycle kit without custom sequencing primer based on the below barcoding strategy (Figure 8). The following read lengths must be used: Read 1: 47 cycles, Index 1: 36 cycles, Index 2: 36 cycles, Read 2: 47 cycles.
    ***Note:*** We suggest to sequence 20–40K reads per cell, but the number of required reads may vary depending on the sample quality.

**Figure 6:**
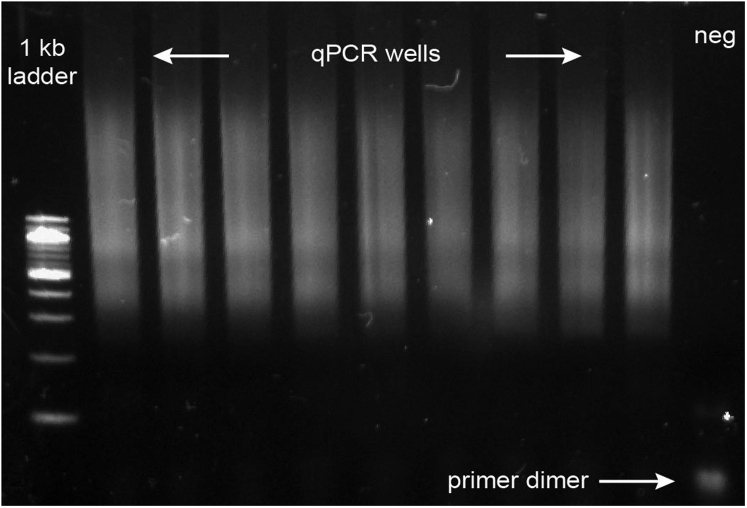
Expected fragmentation patterns from sciATAC-seq protocol qPCR products run on a FlashGel dock to confirm appropriate ATAC-seq library prep. Samples show fragmentation patterns. Negative control shows only a primer dimer and the absence of fragmented DNA. A failed prep would appear as a primer dimer without fragmented DNA.

## Expected outcomes

Following data processing, chromatin accessibility data can be then directly used in ATAC pipelines such as ChromVAR (Schep et al., 2017). For quality control, we propose a first pass pseudobulk check of transcriptional start site (TSS) enrichment using the ENCODE definition of annotated TSSs compared to background (below 8 is a failed library, 8–10 is acceptable, above 10 is good) (https://www.encodeproject.org/atac-seq/). Appropriate nucleosome patterning should also be detectable from aggregated pseudobulk data (Troubleshooting 6). We recommend assessing quality control measures for single-cell data prior to downstream analyses including using cut-offs of fragment of reads in peaks (FRIP) ≥ 0.4 and a minimum of 1000 unique nuclear reads per cell for downstream analyses (Troubleshooting 7). Description of additional analysis tools can be found in LaFave et al. 2020.

## Limitations

We have optimized this combinatorial indexing protocol to work well for multiplexing primary tumor samples, with improvements to avoid FACS sorting and improve fixation and transposition conditions. We recommend using 100K cells for combinatorial indexing with fixation. Low cell numbers may be challenging as there may be cell-type specific loss from fixation and serial washes. Adjustments for cell number should be made at all steps if using a reduced cell number. For example, the fixation step may be omitted to reduce cell loss and fewer transposition reactions may be needed to maintain a ratio of 1,000 cells per reaction. High sample viability at the start of the protocol is important to generate high quality data and cellular dissociation strategies should be optimized for distinct tissue types. There are no stopping points on the first day of the experiment before reverse crosslinking, which can be challenging when coupled with samples requiring long isolation times. Therefore, if sample preparation is long, we suggest viably freezing cells prior to beginning the first day of the protocol.

## Troubleshooting

### Problem 1

In house Tn5 does not have comparable activity to commercial Tn5 (Before you begin).

### Potential solution

An error in the protocol has likely altered the activity of Tn5. In order to identify which step is causing activity loss, it is recommended to assess Tn5 activity by bulk ATAC-sequencing at various steps in the protocol post cell-lysis. For example, perform bulk ATAC-sequencing before and after *E. Coli* precipitation to determine if there was activity loss.

### Problem 2

Cells or transposed nuclei appear to have excess debris (step 3).

### Potential solution

Add in a live-dead sort or dead cell removal approach to improve cell quality. Excess debris can impact cell quality as assessed by quality control metrics like FRIP.

### Problem 3

Cells do not pellet well post-fixation (step 3).

### Potential solution

Confirm that all steps used tubes coated with 7.5% BSA and a bucket rotor centrifuge was used. Primary samples of diverse cell-types can be challenging to pellet without pre-coating tubes with BSA.

### Problem 4

Libraries appear on gel at a lower molecular weight (step 8i).

### Potential solution

This may be indicative of over-transposition of too few cells. Data processing might result in usable libraries; however, increasing the number of cells as suggested in this protocol should overcome this issue.

### Problem 5

Following qPCR, some wells did not amplify product above negative control. qPCR products only show primer dimers when run on a gel (Step 8).

### Potential solution

Errors at several steps could result in loss of material. For example, cell counting may have been inaccurate and fewer cells than expected were added to each well at the beginning of the protocol or at cell splitting. Unequal distribution of cells across well could also be problematic. Confirm accurate cell counting and single-cell resuspension by counting cells at appropriate steps in the protocol.

### Problem 6

Analyzed data appear to have low quality-control scores (step 10).

### Potential solution

Fixation conditions may not have been optimized for sample type. Perform test experiments to optimize fixation conditions from 0.1%–1% to confirm data quality and representation of expected cell types in experiment.

### Problem 7

Few cells pass filter after sample demultiplexing (step 10).

### Potential solution

First, confirm that all experimental quality control checks (agarose gel fragmentation pattern and qPCR) look correct. Next, confirm that appropriate barcodes were used in the demultiplexing part of the protocol. Incorrect barcode assignment can remove high-quality data from the protocol. If barcoding demultiplexing is correct, confirm that peak file is correct (can spot check by assessing peaks in the aggregated .bam file). If an error is not detectable from computational analyses, revisiting debris elimination protocols may be helpful.

## Resource availability

### Lead contact

Further information and requests for resources and reagents should be directed to and will be fulfilled by the lead contact, Lindsay LaFave (lmlafave@mit.edu).

### Materials availability

New materials were not generated for this study.

### Data and code availability

The data and code can be found online at https://www.sciencedirect.com/science/article/abs/pii/S153561082030310X.
